# A framework for quantifying the multisectoral burden of animal disease to support decision making

**DOI:** 10.3389/fvets.2025.1476505

**Published:** 2025-01-23

**Authors:** Sara Lysholm, Gemma L. Chaters, Carlotta Di Bari, Ellen C. Hughes, Ben Huntington, Jonathan Rushton, Lian Thomas

**Affiliations:** ^1^Department of Clinical Sciences, Swedish University of Agricultural Sciences, Uppsala, Sweden; ^2^Animal and Human Health Program, Department of Biosciences, International Livestock Research Institute, Nairobi, Kenya; ^3^Centre for Health Informatics, Computing, and Statistics, Lancaster Medical School, Lancaster University, Bailrigg, United Kingdom; ^4^Lancaster Medical School, University of Lancaster, Lancaster, United Kingdom; ^5^Department of Epidemiology and Public Health, Sciensano, Brussels, Belgium; ^6^Department of Translational Physiology, Infectiology, and Public Health, Ghent University, Merelbeke, Belgium; ^7^Global Burden of Animal Diseases (GBADs) Programme, Liverpool, United Kingdom; ^8^Department of Livestock and One Health, University of Liverpool, Liverpool, United Kingdom; ^9^Royal (Dick) School of Veterinary Studies (R(D)SVS), University of Edinburgh, Edinburgh, United Kingdom

**Keywords:** animal health, disease burden, environmental health, global burden of animal diseases programme, One Health, public health

## Abstract

Animal diseases have wide-ranging impacts in multiple societal arenas, including agriculture, public health and the environment. These diseases cause significant economic losses for farmers, disrupt food security and present zoonotic risks to human populations. Additionally, they contribute to antimicrobial resistance and a range of environmental issues such as greenhouse gas emissions. The societal and ecological costs of livestock diseases are frequently underrepresented or unaddressed in policy decisions and resource allocations. Social cost–benefit analysis (SCBA) offers a comprehensive framework to evaluate the broad impacts of animal diseases across different sectors. This approach aligns with the One Health concept, which seeks to integrate and optimize the health of humans, animals and the environment. Traditional economic evaluations often focus narrowly on profit maximization within the livestock sector, neglecting wider externalities such as public health and environmental impacts. In contrast, SCBA takes a multi-sectoral whole-system view, considering multiple factors to guide public and private sector investments toward maximizing societal benefits. This paper discusses three separate sector specific (Animal health, Human health, Environmental health) methodologies for quantifying the burden of animal diseases. It then discusses how these estimates can be combined to generate multisectoral estimates of the impacts of animal diseases on human societies and the environment using monetary values. Finally this paper explores how this framework can support the evaluation of interventions from a One Health perspective though SCBA. This integrated assessment framework supports informed decision-making and resource allocation, ultimately contributing to improved public health outcomes, enhanced animal welfare, and greater environmental sustainability.

## 1 Introduction

Animal diseases pose multifaceted challenges to human societies, impacting agricultural productivity, public health, and environmental sustainability (1–4). These diseases not only threaten animal health but also have significant economic, social, and ecological consequences. Understanding and quantifying these impacts is essential for informing effective disease management strategies and safeguarding animal and public health as well as environmental sustainability. As major causes of mortalities, poor reproductive performance, limited growth and poor production of, for example, milk and eggs, livestock disease outbreaks lead not only to poor animal welfare, but also substantial losses for farmers and agribusinesses, disrupted supply chains, and undermined food security (5, 6).

In addition, animal diseases pose significant risks to human health, most notably through the transmission of zoonotic diseases. It has been estimated that 60% of human diseases have an animal origin (7, 8), and throughout history, emerging zoonotic diseases have decimated human populations, stirred political change and caused substantial socio-economic declines (9, 10). Concurrently, endemic zoonoses continue to impose a considerable, albeit less obvious, burden, particularly on marginalized livestock-dependent communities through their impacts on both animal and human health (11). Furthermore, antimicrobial resistance, driven by the abundant use of antibiotics in livestock production, poses a growing threat to global health, necessitating integrated approaches to disease control and antibiotic stewardship.

Livestock diseases also impact negatively on the environment, contributing to biodiversity loss, habitat degradation, ecosystem contamination and increased emissions of greenhouse gasses. For instance, diseases in livestock often necessitate increased inputs, such as feed and water, per generated unit of animal-sourced output, and may compel farmers to overstock to compensate for morbidities and mortalities. This, in turn, intensifies pressure on surrounding ecosystems, potentially leading to, for example, deforestation (12) and biodiversity loss (13). Disease outbreaks can lead to increased pressure on natural resources, such as land and water, escalating environmental degradation and threatening ecosystem resilience. Moreover, livestock production systems generate significant greenhouse gas emissions, exacerbating climate change and its associated impacts on human and animal health and wellbeing (14).

Despite considerable animal disease burden, resource allocation for animal health mitigation interventions often do not reflect their magnitude (15–20). One of the problems is that zoonotic and animal diseases have consistently had their societal and environmental burden underestimated (18, 21). Investments in the control of animal diseases, particularly those of commercial livestock, are generally evaluated within economic frameworks with a goal of profit maximization for the livestock sector (22–24). These frameworks tend to focus on farm-level financial analyses and do not generally include externalities, i.e., impacts occurring outside of the production system, such as impacts on human or environmental health or on animal welfare. Instead, regulatory frameworks and taxation or subsidy approaches are used, often without economic estimates to inform their magnitude, to address such market failures in an attempt to optimize societal outcomes (6).

Social cost–benefit analysis (SCBA) is an economic evaluation framework based upon welfare economic theory which attempts to identify and quantify impacts of an activity across multiple relevant sectors of society, attaching a monetary value to as many as possible in order to provide a societal level perspective on investment (25, 26). This evaluation framework has been applied to public sector investments and policy change across infrastructure, education, health and environmental policies (27–31). SCBA can also be used to guide where subsidies and tax abatements could stimulate private sector investment to benefit society (32).

Recently SCBA has been highlighted as a suitable approach for the evaluation of interventions for foodborne and zoonotic diseases (33–36). Optimizing resource allocation according to SCBA with a goal of reaching pareto efficiency is theoretically compatible with the stated aim of the One Health concept as defined by the World Health Organization (WHO) One Health High-Level Expert Panel (OHHLEP): “*to sustainably balance and optimize the health of humans, animals and ecosystems*.” Undertaking a SCBA requires identification both of the costs of an intervention and the impacts of that intervention across relevant sectors. It is therefore imperative that we are able to quantify the burden of disease across multiple sectors which may be averted through our interventions, as well as negative or unintended consequences upon non-target sectors of the intervention itself (37).

Before undertaking any analysis to assess the effect of new interventions that aim to reduce the burden of any disease(s) it is essential to first understand the burden of those diseases across society. Such a burden estimate offers a baseline to support decision making on where interventions are most needed and delineates the maximum theoretical burden that any intervention(s) may avert. The current scarcity of multi-sectoral burden estimates for animal diseases, including foodborne and zoonotic disease, and the need for a standardized methodology and metric to economically evaluate One Health interventions have been the subject of several recent papers (38–40). This paper considers methods applicable to quantifying disease burden and how they may be utilized together to create multi-sectoral burden estimates of the combined societal and planetary burden of animal diseases using monetary values. It illustrates how this framework can support the evaluation of interventions from a One Health perspective though SCBA. The burden framework looks at diseases and injuries of domestic animals and is equally applicable to non-zoonotic animal diseases, zoonoses, foodborne diseases (microbial and chemical) and physical injuries to livestock, including mortality and morbidity due to climatic conditions. These diverse causes are referred to now and throughout as “disease.”

In the paper three domains are considered:

- Human, including physical and mental health and the cost-of-illness attributable to disease;
- Animalia, excluding humans, within which we include the health, wellbeing and productivity of all domesticated (and captive) members of the kingdom; and
- ‘Environment’, within which we consider the abiotic environment, e.g., soil, water and air, and the remaining biota of the earth, being the prokaryotes, bacteria and archaea, and remaining eukaryotes: platae, fungi and protista, and non-domestic members of kingdom Animalia (aka “wildlife”).

These boundary domains are essentially arbitrary and certainly anthropocentric. Humans belong to the family Animalia, and domesticated animals, though contributing >1/5^th^ of global biomass (41) represent only a tiny minority of the identified species in the kingdom Animalia (42). However, despite the principle of equity between sectors attributed by OHHLEP, given that most investment decisions are made through an anthropocentric lens and that burden assessments to date have concentrated predominately on human health and domestic livestock, these boundaries have been chosen for methodological ease.

The paper is divided into the following sections. First, it reviews different methods for estimating the burden of diseases in domestic animals. This is followed by an evaluation of different approaches to estimating the human health burden of animal diseases, and finally the environmental burden of animal disease. For each domain, the data requirements and expected outputs of burden estimates are outlined. A concluding section explores how the different methods can be combined into a joint estimate.

### 1.1 Burden of disease in domestic animals

Current assessments of disease burden in domestic and captive wild animals predominantly hinge upon their economic value to human society and are therefore almost exclusively expressed in monetary terms (20, 24). The economic impact arises from diminished livestock productivity due to mortality, impaired growth and reproductive performance, and reduced yields of products such as milk and eggs, as well as increased expenditures on treatments and control measures, including pharmaceuticals, vaccinations and health care. Wider economic impacts of animal diseases include increased food prices, restricted trade market access and price turmoil following trade and movement bans (supply side shocks), as well as shifting consumer preferences away from animal-sourced food (demand side shocks) (43–47). While the immediate burden of disease is borne by the producers, it later reverberates throughout the entire value chain, affecting other stakeholders such as traders and abattoir workers, as well as consumers (16, 44).

The Global Burden of Animal Diseases (GBADs) program offers a systematic approach to estimating the burden of animal diseases, creating baselines for assessing investments in animal health and production interventions (20, 48, 49). The program has developed an analytical framework that provides a transparent and consistent approach to animal disease burden estimation. This involves a detailed analysis of the “gap” between current livestock production and production in an *optimal* state of health referred to as “ideal.” The approach (50) is applicable across all farmed species and geographical scales (global, national and sub-national) and involves four key stages:

1. Quantifying the biomass and economic value of animals as well as their outputs for the livestock population in the target geographical area (51–53)
2. Estimating the “Animal Health Loss Envelope” (AHLE), a novel metric that calculates the difference in the value of animals, products and expenditures between current realized production and a theoretical “ideal” state without disease, for a specific population of animals over a given time period, thereby capturing both the loss in animals and production and the expenditure in disease mitigation interventions (54, 55)
3. Attributing the AHLE to high-level (infectious, non-infectious and external) or specific disease or syndromic causes, while adjusting for co-morbidities to avoid overestimation of individual disease burdens (55–59)
4. Evaluating the impacts of animal diseases on the wider economy using partial and general equilibrium models, and on gender and human health outcomes (60–62)

Steps 1–3 are farm-level assessments. Step 4 uses information from the previous steps to estimate the animal disease burdens in terms of changes in economic activity measured in gross domestic product (GDP) and economic surpluses.

### 1.2 Data requirements to calculate the animal health loss envelope

Data requirements to calculate the AHLE are extensive and diverse. Identifying the underlying population at risk (for example sheep in pastoral systems in Ethiopia) and appropriate data sources to this population is essential, and ideally, data should be stratified by age-sex groups managed in different systems. A common denominator across species and production systems is necessary for meaningful comparisons, with GBADs using livestock biomass (kg) calculated using data on population size and average live weights. The monetary value of the study population is then estimated using data on population size, production volumes and local market prices.

The economic value and the biomass estimates are integrated into the AHLE model along with data on reproduction (e.g., parturition rate and litter size) and fixed and variable expenditures (e.g., feed, labor, health care). Current production scenarios can be parameterized using information from international databases such as FAOSTAT and WAHIS, national statistics such as census data and figures from meta-analyses or systematic literature reviews. The “ideal,” disease-free scenario can be parameterized through use of biologically plausible maximums, model extrapolations and structured expert elicitation. Once calculated, the AHLE can be attributed to high level causes (infectious, non-infectious and external factors) using structured expert elicitation (56), and to specific aetiological causes or syndromes using data on their prevalence, incidence and production impacts. Disease data can be obtained from systematic reviews and meta-analyses of published and grey literature as well as from national and international statistical bodies, and extrapolation models can be used to address any data gaps (63). When attributing the AHLE, co-morbidities in animals affected by multiple disease must be accounted for, to avoid overestimation of impacts of specific diseases (50).

To estimate the wider economic impact (WEI) of livestock losses, country-specific partial or general equilibrium models are used. These models have complex data requirements needed to simulate a country’s economy. A full account of these data requirements are beyond the scope of this discussion but parameter values are typically sourced from international databases (e.g., FAOSTAT), literature, and the global trade project model (GTAP) databases (64). Outputs from the AHLE models, such as the realized difference in live animals and their produce between current and disease-free scenarios, are used as inputs for these wider economy models, resulting in predicted shifts in market supply, changes in consumer and producer surpluses realized due to improved animal health, and other wider societal externalities.

### 1.3 Human health burden of animal disease

Animal diseases have extensive implications for human health and wellbeing. Zoonotic and foodborne illnesses directly impact on human morbidity and mortality rates, resulting in costs related to treatments and productivity losses, including but not limited to foregone salaried income and inability to attend to crop fields or care for livestock (65). Additionally, animal diseases exacerbate malnutrition outcomes by reducing household production of animal-sourced products or diminishing household revenues, thereby affecting dietary diversity and overall health and economic outcomes (66). The human health burden is often expressed as a measure of utility lost using a variant of a Health Adjusted Life Years (HALY), which combines disease morbidity and mortality impacts into a non-monetary measure. Notable examples include the Quality Adjusted Life Years (QALYs) and Disability Adjusted Life Years (DALYs), both of which are composite metrics incorporating aspects of quantity and quality of life. While alternative HALY metrics, such as the wellbeing-adjusted life year (WALY) (67, 68) and capability-adjusted life year (CALY) (69), have been proposed to better capture additional intangible dimensions of health, QALYs and DALYs remain the most widely used. QALYs are primarily used in evaluations of health care technology and investments (70, 71), while DALYs is the preferred method for disease burden assessments (72). However, while the DALY methodology has undergone refinements in recent years (73), methodological inconsistencies remain a repeatedly highlighted issue warranting consideration (74). Currently, DALY assessments exists for various diseases, injuries and syndromes, including zoonotic diseases such as tuberculosis (75) and brucellosis (76) as well as other syndromes with clear linkages to animal disease such as human malnutrition due to over- or under-consumption of animal source protein (77).

Cost-of-illness (CoI) is a distinct yet closely related concept to the utility-based burden of disease, representing the financial implications associated with a specific illness. It encompasses direct health losses as well as indirect expenses arising from individual and societal responses to the presence or risk of disease. This includes tangible financial outlays and the subjective impact felt by individuals, their families, healthcare providers, and society at large. Compared to utility based HALY metrics, the cost of illness approach is more akin to the AHLE of GBADs, as the expenses span across various domains, including healthcare expenditures such as medical treatments and hospital stays, to non-medical costs like transportation and caregiving as well as productivity losses resulting from missed workdays or reduced productivity due to illness (78–82).

### 1.4 Data requirements for calculating the human health burden resulting from animal disease

Estimating the human health burden caused by diseases in animals using DALYs requires comprehensive data of high quality from multiple sources. Demographic data, such as the total number of males and females per age group and life-expectancy (whether general or local), for the selected area and time period, can be sourced from national statistical institutes or from the United Nation’s Statistics Division (83, 84). Human epidemiological data, such as incidence or prevalence, can be obtained from disease registries, health statistics and scientific and grey literature, and should be stratified by age and sex for more accurate estimates and to facilitate the study of disease burden by these demographics. Stratification by sub-region, occupation and socio-economic status can further enrich data analysis. The severity of health states is incorporated into DALY estimates through data on disease duration and impact (i.e., the “disability weight”), sourced from hospital registries, literature reviews, or expert elicitation, the latter of which often is derived from the Global Burden of Disease (GBD) study (85). Knowing the number of cases in various health states is crucial, and different approaches for obtaining this data have been suggested depending on data availability (86). Data gaps and uncertainties can significantly influence estimates, potentially leading to underestimation or overestimation of disease burden, which can be addressed by using multipliers and extrapolation models based on data from neighboring regions or different time periods and conducting sensitivity analyses (86).

CoI assessments to a large extent require similar information as DALY estimations, including for example demographic and epidemiological data. CoI analysis also necessitate different cost data depending on the analysis perspective. For example, a CoI analysis from the healthcare payer’s perspective includes only direct costs incurred by the payer (e.g., health insurance), while a societal perspective also considers indirect costs and “out-of-pocket” expenses. Direct healthcare costs (e.g., hospitalization and pharmaceutical drugs) can be obtained from healthcare registries, governmental bodies and hospitals, while direct non-healthcare costs (e.g., transport and childcare) can be collected via surveys or expert elicitations. Indirect costs (e.g., productivity losses) depend on the approach used. The human capital approach is commonly applied, where productivity losses are calculated by multiplying the time lost from work by the average daily wage, while special consideration is required to assess productivity losses for individuals unable or unwilling to return to work, for example due to death or severe disability (87).

### 1.5 Existing methods for joint assessment of disease burdens in humans and animals

When considering how to bring disease burdens in the human and animal domains together, much of the existing literature primarily focuses on zoonoses. While numerous studies have separately reported human and animal burdens of zoonotic disease, efforts have been made to integrate the two into a joint metric. An important example is the “zoonotic DALY,” or zDALY, which is a composite HALY metric where the economic impact of animal disease is converted into an “animal loss equivalent” (ALE) which is then added to the human DALY estimate (88). The ALE is derived by quantifying livestock losses and normalizing them against national income values, thereby representing the amount of time required for an average income earner to make up for the economic loss resulting from animal disease (88). The zDALY has been applied to assess the combined human and animal burden of various zoonotic diseases such as brucellosis and rabies (21, 40, 89).

An alternative approach has been proposed by Herrera-Araujo et al. (90), where a monetary value is assigned to the human HALY metric and then combined with the already monetized animal disease burden. While some individuals may morally object to assigning a dollar value to health, monetizing HALY estimates is common practice, particularly in judicial litigations as well as in informing policy decisions and resource allocations (91–95). Different methods exist for determining the “price per HALY,” each of which has its advantages and disadvantages and the choice of methodology should therefore be tailored to the specific context and data availability (96, 97). These methodologies will be discussed later in this paper. Monetizing the HALY burden offers a straightforward pathway for incorporating the cost-of-illness, akin to how the GBADs health-loss-envelope concept includes the cost of responding to disease within livestock. This approach could also be integrated into a SCBA framework together with the monetary burdens of the animal and environment sector.

## 2 Environmental health burden of animal disease

Animal diseases adversely impact the environment through various mechanisms, including increased greenhouse gas emissions resulting from inefficient production (98–101) and environmental degradation following compensatory measures to address disease-related morbidity and mortality, such as increased stocking numbers and densities. Examples include deforestation to create new pastureland, soil degradation, desertification, and eutrophication due to manure run-off into waterways or the damming of streams to generate drinking water for livestock (102, 103). While animal disease can positively impact biodiversity, for example by reducing the number of grazing livestock in an ecosystem and thereby contributing to the preservation of local flora and fauna (REF), they generally have negative effects. For instance, the transmission of diseases like rabies between domestic and wildlife populations can lead to increased mortality, as seen in for example African wild dogs in the Serengeti ecosystem (104), and poor reproductive performance, as observed following *Brucella* spp. Infection in buffaloes (105, 106). Furthermore, human responses to animal diseases can yield unintended environmental consequences. Significant declines in vulture populations in Asia in the late 20th and early 21st centuries have been linked to their scavenging on livestock carcasses containing residues of diclofenac (107), a non-steroidal anti-inflammatory drug (NSAID) widely used in livestock that is highly toxic to several vulture species (108, 109). Reduced vulture populations leave room for species like dogs and rats to scavenge on carcasses, which may increase the spread of diseases such as such as rabies, the bubonic plague and leptospirosis (107, 110). Additionally, certain anthelmintics used in livestock are toxic to various terrestrial and aquatic species (111, 112), negatively affecting insect species such as coprophagous flies and dung beetle larvae (113–115), which hinders grass growth, increases nitrogen volatilization (116) and reduces feed availability for insectivorous species (111).

The ill-defined concept of ideal environmental health is inherently complex and subjective, especially when compared to assessments of health in the human and animal domains. While biota, including sentient biota, are components of every environmental system, the environment itself lacks sentience and cannot be assumed to have a perspective from which its own health can be judged. An understanding of environmental health therefore depends upon the perspective of those evaluating it and the functions they expect it to fulfill. Existing definitions of environmental health often contain concepts such as absence of disease, presence of “stability,” “resilience in the face of stress” (essentially a trait of homeostasis), and “vigor,” which refers to an environmental system’s ability to perform functions or provide services. Consequently, environmental health is a multifaceted and dynamic concept, with various indicators used to assess different terrestrial and aquatic ecosystems (117, 118). Despite the intrinsic value of all environmental systems, much of the work undertaken to assess environmental health focuses on the system’s ability to perform functions that benefit humans, both in terms of natural resources and “environmental services” (119).

From the perspective of domestic livestock production, environmental health burdens have traditionally been considered “externalities” and not included in production costs. Recent advances in our consideration of environmental health have improved how these externalities are measured and quantified. Key approaches include life cycle analysis (LCA), which evaluates potential environmental impacts throughout a product’s life cycle, from raw material acquisition to waste management (120). There are multiple methods and a plethora of environmental indicators which have been used in LCA, but movements toward standardization have been made through ISO and EU standards (121). LCAs analyze the flow of resources through a production cycle and quantify the impact of these product flows on representative indicators across relevant “impact categories” using environmental models. Impact categories can be monetized through shadow pricing, adoption of “full cost accounting for food,” and the incorporation of natural capital accounting into the System of National Accounts (122, 123). Impact categories relevant for studies in livestock include for example acidification, biodiversity, ecotoxicity, greenhouse gas emissions and land use changes (124); however, previous studies have generally focused on greenhouse gas emissions (124–127, 128, 129).

Another method for extrapolating the environmental impact of disease is through the Global Livestock Environmental Assessment Model (GLEAM) developed by the Food and Agriculture Organization of the United Nations (FAO) (130, 131). This model enables calculation of greenhouse gas emissions and nitrogen use per product unit throughout livestock production. It is utilized in the Economics of Ecosystem and Biodiversity (TEEB) project to illustrate the environmental consequences of changing mortality levels in traditional Tanzanian livestock production, which also considers the land-footprint required for feed demand (132). Another method is “Ecosystem Accounting,” which identifies and quantifies the capacity of an ecosystem to provide services such as water purification, flood protection and carbon sequestration. Ecosystem services (and dis-services) provided by livestock production have been studied within the TEEB project, where disparities in carbon sequestration between pastoralism and sedentary beef production in the Maasai steppes has been analyzed (133). The authors monetized the carbon stocks using the USA Interagency Working Group (IAWG) social cost of carbon, which reflects the anticipated damage to the global economy resulting from greenhouse gas emissions effects on climate change (132). This method could be extended to include other environmental impacts in related analyses.

GLEAM, LCA and ecosystem accounting can be used to model “optimal” and “current” scenarios to create an environmental impact envelope equivalent to the AHLE proposed by GBADs. Monetary valuations using willingness to pay (WTP) methodologies or market prices, would then allow this environmental impact to be combined with the animal and human disease burdens to move toward a comprehensive impact assessment of animal disease.

### 2.1 Data requirements for incorporating environmental health into animal disease burden assessments

To quantify the impacts of livestock health on multiple dimensions or measures of environmental health, we propose a framework akin to that used for estimating disease burden in humans and animals. This suggested framework measures the difference between the current and optimized scenario (where livestock are disease-free and production scope, breeds and management remain constant), by quantifying the difference in methane and CO_2_ emissions, land use, acidification, biodiversity, and drug usage related ecotoxicity (anthelmintics, antibiotics, non-steroidal anti-inflammatory and acaricides). During the progression from current conditions to optimal scenarios, interventions may lead to shifts in drug use and initial population increases, necessitating accurate modeling to assess these changes and their environmental impacts. Access to this data could inform policy to maintain steady livestock populations despite reduced disease burden, using economic measures such as taxation or agreements between authorities and livestock keepers, so that multi-sectoral approaches to improved livestock, human and environmental health can be developed.

Data requirements to incorporate environmental health impacts include those already used to populate the dynamic herd models that estimate the AHLE, plus:

1. Methane and CO_2_ emissions (kg) measured per animal per day/week/month/year, stratified by age-sex groups, breed and production system.
2. Production system specific land use per animal (km^2^) per day/week/month/year.
3. Pharmaceutical drugs used per animal (kg) per day/week/month/year stratified by age-sex groups and production systems, at least in groups of antimicrobials, anthelmintics, acaracides, NSAIDs and others, or more specific data if available.

## 3 Using monetary valuations to create a multi-sectoral societal burden assessment for animal disease

The methodologies discussed above for assessing disease burdens across human, animal and environmental domains share certain attributes conducive to a multi-sectoral societal burden assessment. Each domain allows for the modeling of an “optimal or ideal” health scenario and the determination of the gap between this and the current state.

**Figure 1:**
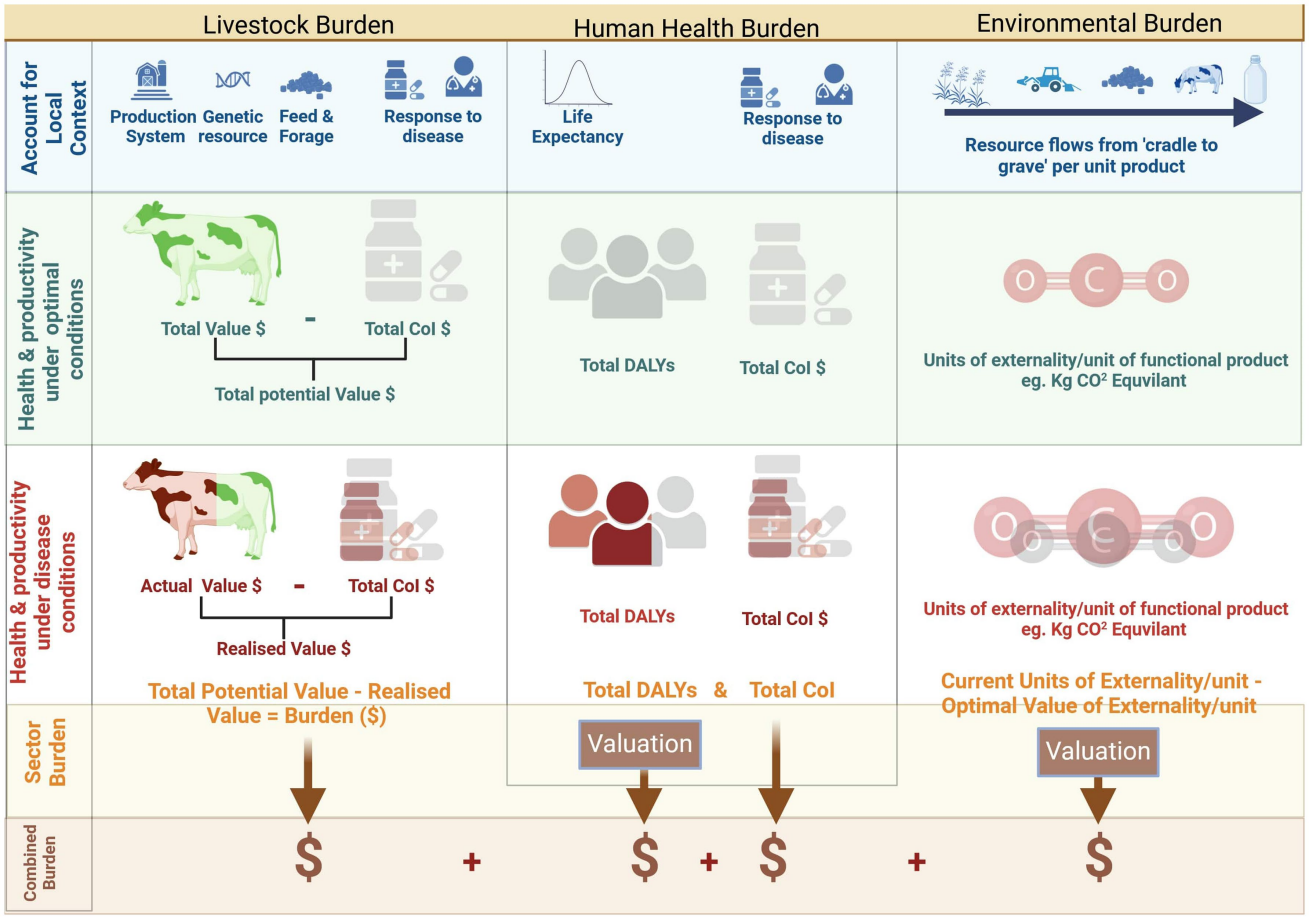
Combining disease burden across animal, human and environmental domains. Created with BioRender.com.

The global burden of human disease is the gap between a state where zero DALYs are attributable to disease and injury and current state, which in 2019 was estimated at 2540 million DALYs (95% uncertainty interval 2,290 m to 2,810 m) (85). The DALYs attributable to animal diseases, such as zoonotic or foodborne disease, or malnutrition due to over- or under-consumption of animal source protein, can be monetized using appropriate methodologies such as WTP as employed by the World Bank (134) and FAO (135), value of statistical life (VSL) or value of statistical life year (VSLY) data, global minimum DALY values, or using national GDP data (21, 90, 95, 136–140). The total human burden attributable to a disease would also encompass the cost-of-illness linked to that disease, carefully ensuring consideration of co-morbidities to avoid double counting. The AHLE (54) constitutes an analogous method for livestock, estimating total burden by calculating differences between the “zero-burden” state and the current scenario in terms of production losses and additional expenditures (equivalent of human CoI). Likewise, an LCA or GLEAM modeling approach, utilizing contextually relevant inputs, would be amenable for a gap-analysis approach, with burden being represented by the difference in monetized environmental externalities between production under ideal conditions and the current state. The combined societal value concept is illustrated in Figure 1.

## 4 Utilization of multi-sectoral burden assessment in social cost–benefit analysis

While the proposed framework aims to facilitate estimations of the multi-sectoral societal burden of animal diseases, its ultimate objective is to support decision making in animal disease control for the benefit of humans, animals and the environment. Currently, decision-making in animal health often relies on cost–benefit, cost-effectiveness and partial budget analyses, but these do not fully capture the multi-sectoral impacts of the diseases of interest (141).

Multi-criteria decision analysis (MCDA) is a semi-quantitative framework which can incorporate different perspectives and multi-sectoral impacts, but if monetary outcomes such as return on investment is required to guide decision making, social cost–benefit analysis (SCBA) may be a more appropriate approach (33, 34). Since 2013, SCBA has been used to evaluate public health policies in the Netherlands, including interventions aimed at reducing the human health burden of the zoonotic protozoic disease toxoplasmosis (35). SCBA extends cost–benefit analysis to better consider costs and benefits across multiple sectors, incorporating the viewpoint of society as a whole (142). SCBA can assist in identifying interventions that yield the largest net social benefit as well as demonstrating which sectors experience net gains and losses, thereby indicating where resource transfers may be required.

Multi-sectoral burden of disease estimates, quantified in monetary units, provide an ideal input for future SCBA analyses of animal disease interventions. Burden estimates create a theoretical maximal envelope of which interventions have the potential to avert a proportion, dependent on their efficacy. Attribution of a predefined burden envelope also reduces the risk of double-counting, or over-inflation of burden averted due to any intervention. Understanding the expenditure in the current state of animal disease, or in future scenarios that may involve an intervention, is not a trivial exercise. Investigation of societal burden requires consideration of the public and private goods generated by (or negatively impacted by) animal health. Data on expenditure would be required from multiple public and private actors, including ministries for livestock, aquaculture, human health and environmental management, as well as education and research sectors. Existing protocols for defining expenditure should be leveraged. The Organization for Economic Cooperation and Development’s (OECD) Classification of the Functions of Government (COFOG) is a broad, high-level assessment, restricted to government spending but across a comprehensive range of sectors (143). System of Health Accounts (SHA-11), established by the International Health Accounts Team (IHAT) of the OECD, WHO and Eurostat, is a more in-depth analysis including public and private costs, yet restricted to the human health sector (144). To facilitate standardized, sustained assessment of expenditure change across the three domains of interest, a protocol for collection of data could be established.

## 5 Limitations of a monetary approach to disease burden

### 5.1 Intangible disease burdens

In addition to the burdens already considered in this paper, there are also a myriad of intangible impacts of animal diseases on society. Related to cases of human disease arising from animal diseases, examples include emotional distress stemming from pain, suffering, anxiety, and grief caused by social limitations imposed by disease (145, 146). Family members, relatives, friends and colleagues may experience distress due to concern for the sick individual and potential social exclusion, particularly if the disease in question is highly infectious or stigmatizing (147). When animals are affected by disease, humans can experience similar pain and anguish, and disease circulation in livestock can give rise to severe emotional distress caused by fear of economic consequences and negative impacts on food security (47, 148, 149), or as reactions to mass culling events as observed during outbreaks of foot and mouth disease (FMD) in the UK and *Mycoplasma bovis* in New Zealand (47, 148–156). Eco-anxiety, i.e., feelings of angst and stress related to the degradation of the natural world also affect emotional wellbeing (157–160), for example, among pastoralists whose livelihoods largely depend on favorable environmental conditions (161). Due to the inherent challenges in quantifying human psychological experiences, intangible costs are frequently left out of CoI assessments, leading to underestimation of the burden of disease (82, 146).

### 5.2 Differential burdens of disease across societal groups

The burden of disease varies across different societal groups. It is crucial to acknowledge these differences to ensure investments in interventions are targeted effectively. The variations in disease burden largely depend on an individual’s contact patterns with animals, animal products and/or excreta, as well as their reliance on animals for livelihood and emotional wellbeing. These contacts are in turn conditional on profession, gender, ethnicity, wealth and educational level (162–167). For instance, pastoralists across Africa, who heavily depend on animals for their livelihoods and on the environment for providing sufficient grazing and water for their livestock, are at high risk of being affected by outbreaks of zoonotic diseases (168). Gender also plays important roles, as women and men traditionally are involved in different household activities and thereby risk exposure to different zoonotic diseases. For example, in many self-sustenance farming societies across the globe, slaughter is a male-dominated activity, while carcass dressing and food preparation generally are considered female tasks (169–172). Furthermore, many argue that the burden of zoonotic diseases is disproportionately borne by women, who often serve as the main caregivers for both sick humans and animals. Due to restricted access to livelihood-generating activities such as land ownership, women are often more dependent on animal husbandry of for example poultry and dairy animals, thereby becoming more vulnerable to the effects of poor health in such species (150, 173–175).

Utilizing a monetary burden of disease inherently requires assigning a value to non-market goods, as the analysis relies on market prices. While certain diseases may appear to have a low burden based on monetary assessments, disproportionate impacts on certain members of society, such as women and children, may be overlooked with this approach. As a result, the framework’s ability to inform advocacy for improved societal outcomes, including equity, may be limited. This underscores the importance of looking beyond purely financial measures when assessing disease burden. Incorporating multi-criteria decision analysis that considers broader societal indicators can provide a more comprehensive basis for decision-making and better address equity and other key outcomes.

### 5.3 Animal-centric burdens of disease

The burden of animal disease discussed in this paper is entirely related to their role as an economic resource to humans, thus overlooking the intrinsic value of animals’ lives from their own perspective. To address this gap, several suggestions of HALY metrics that express disease burden in animals from their own point of view have emerged. Among these, the Animal Life Years Suffered (ALYS) assesses life quality but disregards premature death (176), while the Welfare-Adjusted Life Year (WALY) and Animal Welfare Loss (WAL) incorporate both suffering and premature death, under the premise that sentient animals have a vested interest in their continued existence, and assuming moral equivalence between all animals. Thus far, these metrics have been applied to both food-producing (176) and companion animals (177). Furthermore, the Morally Adjusted Animal Years (MAL) and Species-Adjusted of Suffering Years (SAMY) utilize a species-based modifier to account for either the species degree of self-awareness, or the value ascribed to it by humans, respectively (178). However, these methods have yet to be extensively used in research, and their utility is somewhat constrained by the fact that animal longevity is often determined by their human owners whose aim is to maximize the animal’s production input/output quota rather than increase their life-span (21).

### 5.4 Ethical considerations in applying a monetary value to non-market goods

The suggested framework is based upon a utilitarian, neo-classical economic framework where maximizing the consequential net utility (here considered to be the monetary value) equates to making the “right” ethical decision. WTP methods assume that trade-offs, such as risks to human life, biodiversity loss or animal welfare reduction, can be compensated monetarily. This contrasts with deontological or rights-based ethics, where certain values are intrinsic and not compensable by money (179). The dichotomy between utilitarian and rights-based stances for non-market goods has been demonstrated in a German study among individuals who consider farm animal welfare as important. While high WTP for farm animal welfare was identified among certain individuals, others viewed it as a moral issue, deeming it inappropriate to assign a WTP value (180). Evidence suggests that respondents in WTP surveys often have an incomplete understanding of what a WTP value should include. They may find the concept of valuing non-market goods such as biodiversity an alien concept and, when informed of its use in cost–benefit analysis, might even retract their WTP estimate (181).

Strong critiques of assigning monetary values to human lives highlight the ethical dilemmas at the heart of any resource allocation decision (182–185). While the validity of WTP techniques should be scrutinized, in the absence of a deontological framework for healthcare resource allocation, it is important to continue to improve WTP techniques as they are inevitably going to be utilized within decision making frameworks (179, 183). In the future, it is essential to consider consumer values which may influence WTP, carefully craft WTP surveys to allow participants time to consider presented trade-off dilemmas, and explore the linguistic models used in framing WTP questions (186–188).

### 5.5 Alternative lenses for assessing societal disease burden

When evaluating disease burden through a One Health lens, it becomes evident that the conventional notion that “diseases are inherently negative and should be prevented” oversimplifies a multifaceted reality. For instance, while the presence of the Tsetse fly vectors of human sleeping sickness and Nagana disease in cattle have barred cattle production from large regions in Africa, this has in turn been recognized for its contribution to preserving natural flora and fauna in the same areas (189). Also, while the burden of communicable diseases has been significantly reduced following improvements in human health and living standards in the last centuries, a shift in human disease spectrums from communicable to non-communicable lifestyle diseases, such as obesity and diabetes, has been observed (190, 191). This illustrates that even if the burden of infectious diseases is reduced, humans continue to fall ill, and the burden of disease on our health as well as the healthcare system remains substantial. To acknowledge these intricate relationships between diseases and our societal and planetary health in a transparent manner when conducting disease burden assessments is critical for minimizing collateral damage when formulating intervention strategies.

These limitations, rather than undermine the value of a monetary framework for disease burden, highlight the inadequacy of a single point of view to inform decision-making. Utilizing a SCBA framework to highlight the societal value of certain actions expressed in monetary terms is a valuable tool for supporting decision making, although its use as an isolated approach may be viewed unfavorably (192). All decisions on disease control should be placed within the context of the relevant ethical and legal frameworks at play.

## 6 Conclusion

Animal diseases impose substantial and diverse impacts on agriculture, public health, and the environment. These impacts, while significant, are often inadequately represented in policy planning and resource allocation decisions. SCBA provides a multi-sectoral evaluation framework that captures the wide-ranging effects of animal diseases and guides investments toward maximizing societal benefits. By integrating SCBA with the One Health approach, it is possible to address the interconnected health needs of humans, animals, and the environment. To effectively apply SCBA and optimize resource allocation, it is crucial to develop standardized methodologies for quantifying the multi-sectoral burdens of animal diseases. Implementing these standardized methods will enable more comprehensive and effective disease management strategies that enhance human and animal health as well as ensure environmental sustainability. Through a coordinated effort that leverages SCBA and One Health principles, we can better understand and mitigate the multifaceted impacts of animal diseases, thereby promoting global health resilience and sustainability.
